# Reference gene selection for quantitative real-time PCR analysis in virus infected cells: SARS corona virus, Yellow fever virus, Human Herpesvirus-6, Camelpox virus and Cytomegalovirus infections

**DOI:** 10.1186/1743-422X-2-7

**Published:** 2005-02-10

**Authors:** Aleksandar Radonić, Stefanie Thulke, Hi-Gung Bae, Marcel A Müller, Wolfgang Siegert, Andreas Nitsche

**Affiliations:** 1 Charité – CCM, Medizinische Klinik II m.S. Hämatologie/Onkologie, Berlin, Germany; 2 Robert Koch Institut, ZBS 1, Berlin, Germany

## Abstract

Ten potential reference genes were compared for their use in experiments investigating cellular mRNA expression of virus infected cells. Human cell lines were infected with Cytomegalovirus, Human Herpesvirus-6, Camelpox virus, SARS coronavirus or Yellow fever virus. The expression levels of these genes and the viral replication were determined by real-time PCR. Genes were ranked by the BestKeeper tool, the GeNorm tool and by criteria we reported previously. Ranking lists of the genes tested were tool dependent. However, over all, β-actin is an unsuitable as reference gene, whereas TATA-Box binding protein and peptidyl-prolyl-isomerase A are stable reference genes for expression studies in virus infected cells.

## Background

Quantitative real-time PCR (QPCR) has become the favoured tool in mRNA expression analysis and also in virus diagnostics [1]. Real-time PCR has outperformed classical and semi-quantitative PCR methods in terms of accuracy, reproducibility, safety and convenience for the precise monitoring of viral load in clinical material, as well as for the investigation of the expression of cellular genes in response to virus infection. However, the most prominent problem in quantitative mRNA expression analysis is the selection of an appropriate control gene. For years, the glyceraldehyde 3-phosphate dehydrogenase (GAP) gene and the β-actin (Act) gene were used as control genes in classical molecular methods for RNA detection. Recently, evidence accumulated that especially these two genes, GAP and Act, are unsuitable controls in quantitative mRNA expression analysis due to setting dependent variations in expression [2-4]. Recently, we have confirmed these results by investigating the expressional stability of 13 potential reference genes in 16 different tissues and presented more suitable genes like the RNA polymerase II gene [5]. However, an evaluation of reference genes in virus infected cells has not been performed so far. Therefore, the selection of the 10 most promising reference genes, GAP, Act, peptidyl prolyl isomerase A (PPI), glucose 6-phosphate dehydrogenase (G6P), TATA-Box binding protein (TBP), β2-microglobulin (β2M), α-tubulin (Tub), ribosomal protein L13 (L13), phospholipase A2 (PLA) and RNA polymerase II (RPII) were evaluated in cell lines infected with members of different virus families: coronavirus (SARS-coronavirus), flavivirus (yellow fever virus, (YF)), herpesvirus (Human herpesvirus-6 (HHV-6) and cytomegalovirus (CMV)) and orthopoxvirus camelpox (CAMP), covering also DNA and RNA viruses. Quantification of viral RNA was performed to proof and monitor infection. Thereafter the candidate reference genes were evaluated by the BestKeeper tool [6], the GeNorm tool [7] and the algorithm we described previously [5].

## Results

An efficient infection could be evidenced by a significant increase of viral RNA or DNA for all 5 viruses over time (table 1). Despite progressing viral replication, the expression of some of the reference genes remained constant, while other genes were varying in expression according to accumulation of infected cells.

**Table 1:** Cell culture conditions and results of virus kinetics

	CMV	HHV-6	CAMP	SARS	YF
cell line	MRC-5	CCRF-HSB-2	HepG2	Huh-7D12	HepG2
multiplicity of infection	2.0	0.5	0.5	1.0	0.5
time to maximal infection /h	72	120	24	72	96
max. infected cells %	100	>70	>90	>70	>80
measuring point /h	0,6,12,24,48,72	0,24,48,72,96,120	0,1,3,6,12,24	0,2,4,22,42	0,24,48,72,96

The experimentally obtained data for each virus and each gene were analysed using three different methods. The reference gene evaluation of the BestKeeper tool is shown in table 2. A low standard deviation (SD) of the C_T _values should be expected for useful reference genes and a high SD for genes that are susceptible to virus replication. Corresponding to the recent estimation the SD of the C_T _value was highest for Act in 4 of 5 viruses, indicating that Act is no reliable reference gene in this setting. In contrast, TBP and PPI displayed the highest expressional stability for 4 of 5 viruses. To find a general conclusion, the total of all SD values from all virus experiments (sum_V_) was calculated for each reference gene. As shown in table 2, TBP and PPI seemed to be the least regulated genes in this analysis (sum_v _= 2.29 for both), followed by GAP (sum_v _= 3.49) and β2M (sum_v _= 3.96). All other genes showed moderate total SD values (sum_v _> 4.58), except Act (sum_v _= 11.28), confirming to be the most inappropriate reference gene. It is remarkable that the obtained BestKeeper index values are low, despite the inclusion of Act in the calculation. Calculating BestKeeper vs. each reference gene using *Pearson correlation *displayed very inconsistent results (table 3). Act showed the highest SD values in all virus infections, but a significantly high correlation. In contrast TBP displayed low correlation that was statistically not significant in most cases. When summing up the SD values of all reference genes for each virus infection (sum_HRG_), it seems that CAMP infection caused the highest variations in reference gene expression.

**Table 2:** Results from BestKeeper analysis, SD [±C_T_]

	RPII	Act	β2M	L13	PLA	TBP	GAP	PPI	G6P	Tub	*BK*	*sum*_*RGC*_
CMV	0.59	2.70	0.51	0.36	0.72	0.41	0.66	0.43	0.71	0.69	0.56	7.78
HHV-6	2.77	1.09	0.50	0.87	0.88	0.35	0.59	0.26	0.92	0.78	0.63	9.02
CAMP	1.84	2.70	1.46	2.34	1.72	0.49	0.61	0.70	1.47	1.36	1.10	14.70
SARS	0.39	1.72	0.41	0.53	0.58	0.32	0.56	0.34	0.81	0.55	0.40	6.21
YF	1.36	3.06	1.07	0.67	1.64	0.71	1.08	0.56	0.80	1.19	0.98	12.16

*sum*_*V*_	6.95	11.28	3.96	4.77	5.55	2.29	3.49	2.29	4.71	4.58		

**Table 3:** Results from BestKeeper analysis, Bestkeeper vs. Reference gene candidate

Coeff. of corr. [r] (*p*-Value)	RPII	Act	β2M	L13	PLA	TBP	GAP	PPI	G6P	Tub
CMV	0.75	0.79	0.76	0.13	0.89	0.10	0.92	0.91	0.75	0.95
	(0.005)	(0.002)	(0.005)	(0.698)	(0.001)	(0.763)	(0.001)	(0.001)	(0.005)	(0.001)
HHV-6	0.79	0.73	0.54	0.30	0.93	0.79	0.94	0.75	0.82	0.97
	(0.002)	(0.007)	(0.069)	(0.350)	(0.001)	(0.002)	(0.001)	(0.005)	(0.001)	(0.001)
CAMP	0.91	0.18	0.98	0.95	0.99	0.78	0.63	0.99	0.45	0.59
	(0.002)	(0.662)	(0.001)	(0.001)	(0.001)	(0.022)	(0.092)	(0.001)	(0.268)	(0.127)
SARS	0.48	0.77	0.41	0.27	0.85	0.88	0.46	0.36	0.73	0.84
	(0.162)	(0.010)	(0.236)	(0.452)	(0.002)	(0.001)	(0.177)	(0.307)	(0.017)	(0.002)
YF	0.90	0.91	0.96	0.25	0.98	0.94	0.99	0.92	0.93	0.92
	(0.001)	(0.001)	(0.001)	(0.492)	(0.001)	(0.001)	(0.001)	(0.001)	(0.001)	(0.001)

Abbreviations: SD [± C_T_]: the standard deviation of the C_T_; *BK*: *BestKeeper*; Sum_V_: Sum of viral infection SD values; Sum_RGC_: Sum of reference gene SD values

Analysing the expression data with the GeNorm tool showed slightly deviant results (table 4). First, the value sum_V_, representing the SD of a reference gene over all viruses, was lowest for PPI (sum_V _= 6.08) confirming the results obtained by the Bestkeeper tool. However, β2M (sum_V _= 6.11), GAP (sum_V _= 6.19) and TBP (sum_V _= 6.29) turned out to be comparably reliable as reference genes. Second, also the GeNorm tool showed that Act is by far the worst reference gene (sum_V _= 14.20).

**Table 4:** Results from GeNorm analysis (M ≤ 0.5)

	RPII	Act	β2M	L13	PLA	TBP	GAP	PPI	G6P	Tub	*sum*_*RGC*_
CMV	1.41	3.41	1.42	1.63	1.45	1.69	1.38	1.37	4.79	1.54	20.09
HHV-6	2.82	1.38	1.15	1.55	1.19	1.03	0.95	1.08	1.15	0.96	13.27
CAMP	1.70	3.84	1.40	1.94	1.49	1.57	1.66	1.40	2.04	1.92	18.95
SARS	0.83	1.88	0.82	1.06	0.87	0.70	0.89	0.84	1.04	0.80	9.73
YF	1.65	3.69	1.32	1.87	2.02	1.30	1.31	1.39	1.31	1.48	17.34

*sum*_*V*_	8.41	14.20	6.11	8.05	7.03	6.29	6.19	6.08	10.33	6.70	

Abbreviations: Sum_V_: Sum of viral infection GeNorm values; sum_RGC_: sum of reference gene GeNorm values

Applying the calculation mode presented previously [5], that is based on the calculation of ΔΔC_T _values (table 5), Act was most susceptible to virus infection for 3 of 5 viruses and displayed the highest ΔΔC_T _value over all viruses (sum_V _= 45.23). The two genes with the lowest ΔΔC_T _value were TBP (sum_V _= 9.82) and PPI (sum_V _= 10.04), corresponding to the results of the Bestkeeper and the GeNorm tool.

**Table 5:** Results from ΔΔC_T _analysis

	RPII	Act	β2M	L13	PLA	TBP	GAP	PPI	G6P	Tub	*sum*_*RGC*_
CMV	2.10	11.55	3.03	2.18	3.95	2.36	2.90	2.54	12.51	2.39	45.49
HHV-6	5.98	3.54	3.35	2.89	4.99	0.88	2.27	1.25	3.35	2.30	30.78
CAMP	3.59	14.19	3.94	3.17	2.71	1.23	3.19	1.78	2.22	3.33	39.33
SARS	1.19	1.71	2.14	1.93	2.52	1.11	2.75	1.34	4.14	1.78	20.58
YF	9.01	14.25	5.78	2.90	9.62	4.24	6.35	3.14	5.42	7.48	68.17

*sum*_*V*_	21.87	45.23	18.22	13.07	23.78	9.82	17.45	10.04	27.62	17.27	

Abbreviations: sum_V_: sum of viral infection values; sum_RGC_: sum of reference gene values

## Discussion

To date, it is generally accepted, that the selection of the ideal reference gene in gene expression analysis has to be done for each individual experimental setting by evaluating several genes and using the best two or three of these genes as reference. Obviously there is no "one good gene for all experiments" recommendation. However, it is helpful to find putative candidates that can be shortlisted when setting up a new experimental design. Therefore, we determined the expression of previously tested reference genes in a setting of virus infected human cell lines. Capable reference genes were evaluated using three independent methods: Bestkeeper, GeNorm and the ΔΔC_T _method, and their results were compared.

All three tools ranked actin at the last position, indicating that it is an unsuitable reference gene in virus infected cells. The actin gene shows significant variations with increasing degree of infection.

The best genes obtained from all three calculation tools were TBP and PPI. TBP seems to be a relative stable expressed gene during the course of virus replication of different viruses in different cells. However, as previously shown [5] TBP is not expressed in all tissues and therefore its use may be limited.

Interestingly, classical reference genes like β2M and GAP were also acceptable regarding to a stable expression in virus infected cells. All other genes showed moderate expression stability.

The analysis of our data set according to the Bestkeeper tool revealed very good BestKeeper indices; even actin was included into our gene panel. These findings demonstrate the usefulness of analysing a wide variety of reference gene candidates. The inconsistent data regarding to the Bestkeeper calculation of the coefficient of correlation and the corresponding p-values may be a result of the *Pearson correlation*. As described by Pfaffl *et al*. its use is limited to groups without heterogeneous variances, but the tested reference genes have very different expression levels resulting in significant variances. Paffl *et al*. also described that new versions of Bestkeeper should circumvent these problems by use of *Sperman *and *Kendall Tau correlation*. However, one problem still remains to be solved; both tools, the BestKeeper and the GeNorm, can not compare paired probes. This is the great advantage of the ΔΔC_T _method, or any other method which directly compares paired samples. From this point of view the use of a method like the ΔΔC_T _should be applied first before considering additional tools for further elucidation of the acquired data.

## Conclusions

In summary, TBP and PPI turned out to be the best reference genes in virus infected cells. These genes are a good point to start reference gene selection in gene expression studies in virus infection experiments.

## Material and Methods

### Virus culture and virus detection by real-time PCR

Camelpox strain CP-19, CMV strain AD169, HHV-6 strain U1102, SARS coronavirus strain 6109 and YFV strain 17D were propagated according to standard procedures [8-10].

The respective MOI and time of cell culture are shown in table 1 and were chosen to allow maximal infection as determined by immunofluorescence and real-time PCR [8-11]. For kinetic studies, cells were harvested at several time points (table 1) and RNA was extracted. The RNA transcription level of putative reference genes was determined by quantitative real-time PCR as described below.

### Extraction of RNA

Total RNA from 1 × 10^6 ^cells was prepared using the QIAamp RNA Blood Mini Kit and RNase-free DNase set (Qiagen, Hilden, Germany) according to the manufacturer's recommendations for cultured cells. RNA solution was treated with DNA-*free *(Ambion, Huntingdon, United Kingdom).

### cDNA synthesis

cDNA was produced using the Superscript III RT-PCR System (Invitrogen, Karlsruhe, Germany) according to the manufacturer's recommendations for oligo(dT)_20 _primed cDNA-synthesis. cDNA synthesis was performed using 1 μg of RNA, at 50°C. Finally, cDNA was diluted 1:5 before use in QPCR.

### Quantitative TaqMan PCR

Primers, TaqMan probes and QPCR conditions for reference gene analysis were used as previously described [5]. PCR was performed in a Perkin Elmer 7700 Sequence Detection System in 96-well microtiter plates using a final volume of 25 μl.

### Calculations

Analysis was performed with the BestKeeper [6] and GeNorm [7] tools. The ΔΔC_T _value was calculated as follows: First the ΔC_T _for each time point of probe assessment between virus and Mock infected cells was calculated. In a second step the maximal differences between the time points were calculated as ΔΔC_T_.

## Competing interests

The author(s) declare that they have no competing interests.

## Authors' contributions

AR conceived the study, carried out the HHV-6 experiments and real-time PCR assays and drafted the manuscript. ST carried out the CMV experiments. HB carried out the YF experiments. MM carried out the SARS experiments. WS participated in the design of the study. AN carried out the CAMP experiments, participated in design and coordination of the study and helped to draft the manuscript. All authors read and approved the final manuscript.
